# The expression and biological role of complement C1s in esophageal squamous cell carcinoma

**DOI:** 10.1515/biol-2022-0915

**Published:** 2024-07-24

**Authors:** Ruomu Ge, Zhengyun Luan, Ting Guo, Sheng Xia, Jun Ye, Jie Xu

**Affiliations:** Central Laboratory, The Affiliated Taizhou People’s Hospital of Nanjing Medical University, Taizhou School of Clinical Medicine, Nanjing Medical University, Taizhou, Jiangsu, 225300, P.R. China; Department of Clinical Laboratory, The Affiliated Taizhou People’s Hospital of Nanjing Medical University, Taizhou School of Clinical Medicine, Nanjing Medical University, Taizhou, Jiangsu, 225300, P.R. China; School of Medicine, Jiangsu University School, Zhenjiang, Jiangsu, 212000, P.R. China; Anhui Province Key Laboratory of Infectious Diseases, The First Affiliated Hospital of Anhui Medical University, Hefei, China

**Keywords:** esophageal squamous cell carcinoma, C1s, proliferation, apoptosis

## Abstract

The present work focused on investigating the role of the altered expression of complement C1s in proliferation and apoptosis of esophageal squamous cell carcinoma (ESCC) cells and explore its biological functions in ESCC, so as to lay a theoretical foundation and provide certain clinical reference for diagnosing and treating ESCC. Complement C1s expression within ESCC was assessed, and its clinical pathological characteristics in ESCC patients were analyzed. Subsequently, *in vitro* experiments were performed to further explore the mechanisms by which complement C1s affected ESCC. According to the results, complement C1s expression within ESCC markedly increased relative to adjacent non-cancerous samples. High C1s expression showed positive relation to race, residual lesion, and tumor location of ESCC patients. Complement C1s affected ESCC cell proliferation and apoptosis. Notably, C1s knockdown significantly inhibited ESCC cell proliferation and enhanced their apoptosis. C1s suppressed ESCC cell proliferation via Wnt1/β-catenin pathway and promoted their apoptosis through modulating the expression of Bcl2, Bax, and cleaved-caspase3.

## 1 Introduction

Esophageal cancer (EC) accounts for a major factor inducing cancer-associated mortality globally, which can be divided as esophageal squamous cell carcinoma (ESCC) and esophageal adenocarcinoma [1]. The diagnosis of EC is usually made at the advanced stage, with a 5-year survival rate of EC patients in China being less than 5% [2,3]. The primary pathophysiological pathogenesis of ESCC is characterized by esophageal damage caused by carcinogenic compounds in direct contact with the esophageal mucosa [4]. Additionally, there are certain genetic factors for ESCC. Three whole-genome association studies are summarized and the new susceptibility loci for ESCC have been identified [5]. The tumor-node-metastasis classification formulated by the International Union for Cancer Control has been widely used to guide the staging of ESCC, which divides the esophagus into four layers: mucosa, submucosa, muscularis propria, and adventitia. The primary tumor (T) staging is based on the anatomical extent of these layers [6].

As shown in some studies, after endoscopic or surgical treatment, early-stage cancer has significantly increased the 5-year survival rate by 80–90% [7]. Therefore, it is crucial to screen for asymptomatic ESCC and early-stage ESCC [8,9]. However, modeling studies indicate that population-based screening for high-risk groups is cost-effective [10].

Complement C1s, a component of the C1 complex, constitutes a part of the innate immune response in the immune system [11]. In the C1 complex, C1s functions as an enzyme crucial for the activation of the immune system [12]. When the C1 complex binds to antigens, C1s will be activated, which triggers a series of immune responses, including processes such as inflammation and antigen dissolution, thus protecting the body from infection [13]. The role of C1s in this process involves activating other complement proteins, such as C4 and C2, to form the C3 convertase, thereby initiating the immune response cascade [14]. Collectively, the complement system is the first-line immune defense against pathogens, and C1s in the C1 complex exerts a critical effect on initiating immune response to external threats [15]. Typically, the C1 complex of complement has long been considered as a vital component of the host defense against microorganisms. And it is also known to recognize subunit C1q, which can directly bind to pathogens or assist in the recognition of pathogens by antibodies or C-reactive protein [12]. Moreover, each component of the C1 complex can exert functions beyond complement activation, like enhancing tumor cell evasion of immune responses, inducing angiogenesis, and promoting tumor proliferation, migration, and invasion [16,17]. Complement components secreted by tumor cells are associated with adverse clinical pathological characteristics, making them the potential biomarkers for tumor diagnosis, classification, and prognosis, or the predictors of tumor invasion and metastasis risk [18]. While the role of C1s in tumors such as skin squamous cell carcinoma, glioblastoma, and urothelial carcinoma has been confirmed, its role in ESCC remains unclear [17,19,20].

In recent years, second-generation and high-throughput sequencing techniques are rapidly developed, sequencing has become a routine experimental modality. Genomic variations primarily result from changes in the base pairs on the DNA sequence, and they exert an important effect on the process of tumor initiation and progression [21]. Researchers have carried out genome-wide association studies, with high-density genetic markers (like single nucleotide polymorphisms or copy number variations) being used for the genotyping of large populations of DNA samples, and established multiple public databases for relevant studies. The goal is to identify genetic factors associated with complex diseases by genetic research methods, thus shedding novel lights on disease occurrence, development, and treatment, and laying an important theoretical foundation for further research [22,23].

We examined C1s expression within EC with The Cancer Genome Atlas (TCGA) database, and explored its expression in ESCC tumor tissues through immunohistochemical and high-throughput sequencing analyses. In addition, the biological effects of C1s on ESCC cells were investigated at the cellular level through *in vitro* experiments. This series of studies aimed to further explore the effect of C1s on ESCC, thus providing new theoretical foundations and strategies for the occurrence, development, and treatment of ESCC.

## 2 Materials and methods

### 2.1 Source and processing of patient tissue data

Three patients with ESCC were selected from Taizhou People’s Hospital, and one postoperative tumor tissue sample and one adjacent non-cancerous tissue sample were obtained from each of the patient, resulting in six samples in total. All the fresh tissues were preserved in the −80°C ultra-low temperature freezer.


**Informed consent:** Informed consent has been obtained from all individuals included in this study.
**Ethical approval:** The research related to human use has been complied with all the relevant national regulations, institutional policies and in accordance with the tenets of the Helsinki Declaration, and has been approved by the Ethics Committee of Taizhou People’s Hospital. Acquisition of tissue samples was granted ethical clearance by Ethics Committee of Taizhou People’s Hospital (approval reference number, KY 2021-084-01).

### 2.2 Cell culture

The cryopreserved human esophageal normal HET-1A cells and the human EC TE-2 cells were first retrieved from liquid nitrogen, prior to immediate placement in the 37°C water bath for thawing. Afterward, cells were cultured in the complete growth medium. To be specific, HET-1A cells were cultivated in high-glucose Dulbecco’s modified eagle medium (Solarbio, Beijing) that contained 10% fetal bovine serum and 100 U/mL penicillin–streptomycin (Solarbio, Beijing), while TE-2 cells were cultivated in RPMI-1640 (Solarbio, Beijing) that contained 10% fetal bovine serum and 100 U/mL penicillin–streptomycin. Cells at logarithmic phase were harvested to conduct transfection. Cell density was adjusted on the day before transfection, then cells were evenly inoculated into the 24-well plate (containing 500 μL single-cell suspension per well), followed by overnight incubation at 37°C with 5% CO_2_. At 4 h prior to transfection, the culture medium in the 24-well plate was replaced with the antibiotic-free medium. Using the RFect nucleic acid transfection reagent, cell transfection was done according to specific instructions. In brief, 6 pmol siRNA and 50 μL serum-free medium were gently mixed in a 1.5 mL EP tube, while 50 μL serum-free medium as well as 2 μL RFect was gently blended in another 1.5 mL EP tube. After 5 min standing under ambient temperature, we blended siRNA-containing medium with RFect-containing medium, and left the mixture stand for additional 20 min under ambient temperature. Subsequently, the mixed liquid was dropped into the 24-well plate under gentle shaking to ensure even mixing. Later, the plate was cultivated at 37°C. After 4–6 h, fresh complete growth medium was added to replace the original one for subsequent experiments.

### 2.3 Western blot

When reaching 80% cell confluence in a six-well plate, cell lysis buffer (200 μL) that contained protease inhibitor (cell lysis buffer: protease inhibitor = 100:1; Beyotime, Shanghai) was added. After 20 min incubation on ice, the cell lysis buffer was blown down, then the lysate was transferred into an EP tube and centrifuged (4°C, 12,000 rpm × 15 min). The BCA assay (Beyotime, Shanghai) was conducted to measure the protein content. We later introduced loading buffer into the EP tube, mixed sufficiently, and protein was boiled for 10 min. Also, the 10% separating gel and 5% stacking gel were prepared. Later, 1× SDS-PAGE electrophoresis solution was loaded, the comb was slowly removed, 3 μL marker was added, and then 20 μL protein sample was introduced for electrophoresis. For blotting, the polyvinylidene fluoride (PVDF) membrane (Merck, Germany) was prepared in advance in methanol for 2 min. After electrophoresis, the gel was arranged, the paper was filtered, and the pad was sponged sequentially from the negative electrode to the positive electrode (sponge → filter paper → gel → PVDF membrane → filter paper → sponge), with removal of air bubbles between the gel and the membrane. Then, the membrane was placed in the tank, and the transfer solution was added to transfer at 200 mA on ice for 2 h. After blotting, the PVDF membrane was immersed in 5% defatted milk for a period of 1 h under room temperature, and rinsed thrice with 1× Tris-Buffered Saline Tween-20 (TBST) (10 min each), followed by 1 h incubation using the diluted primary antibody (Abcam, UK) under ambient temperature. The membrane was later rinsed thrice with 1× TBST (10 min each), and further incubated with the diluted secondary antibody (Abcam, UK) for a 1 h period under ambient temperature. Following washing thrice with 1× TBST (10 min each), equal volumes of the freshly prepared luminescent liquids A and B were mixed, the instrument was exposed for imaging, and the picture was saved.

### 2.4 Cell counting kit-8 (CCK-8) assay

Following 24 h transfection, cells (5,000/well 100 μL medium) were inoculated into the 96-well plate, along with non-transfected cells. Following 24 h incubation, CCK-8 solution (10 μL, Thermo Fisher, USA) was introduced into each well. The plate was then subjected to additional 1.5 h incubation. Afterward, the 96-well plate was taken out, and the optical density values were detected with the microplate reader at 450 nm. Every assay was carried out thrice. The data were collected for statistical analysis.

### 2.5 Flow cytometry

After transfection, Annexin V-PE Apoptosis Detection Kit (Thermo Fisher, USA) was employed for cell detection. Specifically, cells in each group were digested with trypsin (Solarbio, Beijing) and centrifuged at room temperature at 1,200 rpm for a 5 min duration. Later, cells were resuspended into pre-chilled phosphate buffered saline (PBS), centrifuged again at room temperature, and the supernatant was carefully removed with a gun. After resuspension again with 1× binding buffer, the cell density was adjusted to 1 × 10^6^ cells/mL. Afterward, 100 μL of cells to be tested were placed in the flow tube, then Annexin V-PE and 7-ADD (5 μL each) were introduced to incubate the cells for 30 min in dark under ambient temperature (note: cells were dispersed to avoid clumping). Later, 1× binding buffer (400 μL) was introduced and mixed sufficiently, the mixture was placed on ice and tested within 1 h. Flow cytometry was performed to measure cell apoptosis. Notably, the lower left and right quadrants stand for viable and early apoptotic cells, separately; whereas the upper left and right quadrants represent dead and late apoptotic cells, respectively. Data were analyzed using Flowjo software.

### 2.6 Immunohistochemical staining

(1) Antigen retrieval: Tissue sections were put into citrate buffer (pH 6.0) to heat until boiling. Once boiling, the sections was steamed for 3 min, then the heat was turned off and the sections were cooled naturally to ambient temperature. PBS was then added to wash the sections thrice for 5 min.
(2) Blocking endogenous H_2_O_2_: The sections were washed under running water, soaked in distilled water, and placed in the freshly prepared 3% H_2_O_2_ solution for 20 min in dark. Later, the sections were rinsed again with PBS buffer for 3 min twice.
(3) Blocking: Following blocking, sections were placed in an incubation box. A dedicated immunohistochemistry pen (PAP pen) was used to circle the tissue on the sections to prevent antibody overflow during the experiment.
(4) Primary antibody incubation: The primary antibody (anti-hC1s) was diluted at 1:200, and applied to completely cover the tissue on the slides, prior to overnight incubation under 4°C.
(5) Rewarming: Slides were taken out of the refrigerator, rewarmed under ambient temperature for 10–30 min, and rinsed with PBS buffer for 5 min thrice.
(6) Secondary antibody incubation: The secondary antibody diluted at 1:1,000 was added to further incubate the tissue on the slides in the 37°C incubator for 15 min.
(7) PBS washing: Slides were rinsed with PBS buffer for 5 min thrice.
(8) 3,3′-Diaminobenzidine (DAB) staining: The freshly prepared DAB staining solution (50 μL/slide) was added to the incubator. The staining time was adjusted according to the tissue staining intensity. After staining, slides were rinsed thoroughly with running water for 10 min.
(9) Counterstaining: The tissue was counterstained with hematoxylin for 60 s, and then washed under running water.
(10) Dehydration and drying
(11) Mounting and observation: Slides were mounted using neutral gum before observation with a microscope.

### 2.7 Real-time polymerase chain reaction (PCR)

The RNA extraction kit (Thermo Fisher, USA) was employed for extracting total RNA, which was then prepared into cDNA with the cDNA synthesis kit (Thermo Fisher, USA) and used as a template in the PCR system. The reaction system (10 μL in volume) was gently mixed by flicking, and then centrifuged.

**Table:** 

EvaGreen 2× qPCR MasterMix	5 μL
Forward primer (10 μm)	0.3 μL
Reverse primer (10 μm)	0.3 μL
Template DNA	Variable (≤500 ng/reaction)
Nuclease-free H_2_O	To 10 μL

The PCR procedure was conducted on the PCR machine (Roche, LightCycler480). To be specific, the PCR procedure is shown as, 10 min under 95°C; then 15 s under 95°C and 60 s under 60°C for 40 cycles. A melting curve analysis was performed, and the data were exported. The final analysis was conducted using the 2^ΔΔCt^ method.

### 2.8 High-throughput sequencing

High-throughput sequencing was carried out for tumor tissues and the matched non-cancerous samples were harvested in three patients with ESCC. Raw data were aligned to reference genome with BWA software, sorted with Samtools, and duplicates were removed by Picard. Subsequently, the sequencing results were thoroughly analyzed, and a heatmap was plotted with ggplot2 for visualization.

### 2.9 Statistical analysis

Statistical analysis was conducted by employing GraphPad Prism 8.0 statistical software. Measurement data were indicated by mean ± standard deviation. A *t*-test was applied in comparing two groups, whereas one-way ANOVA analysis was employed to compare several groups. *P* < 0.05 stood for statistical significance.

## 3 Results

### 3.1 Complement C1s expression within ESCC is markedly up-regulated relative to normal tissues and adjacent non-cancerous tissues

**Figure 1:**
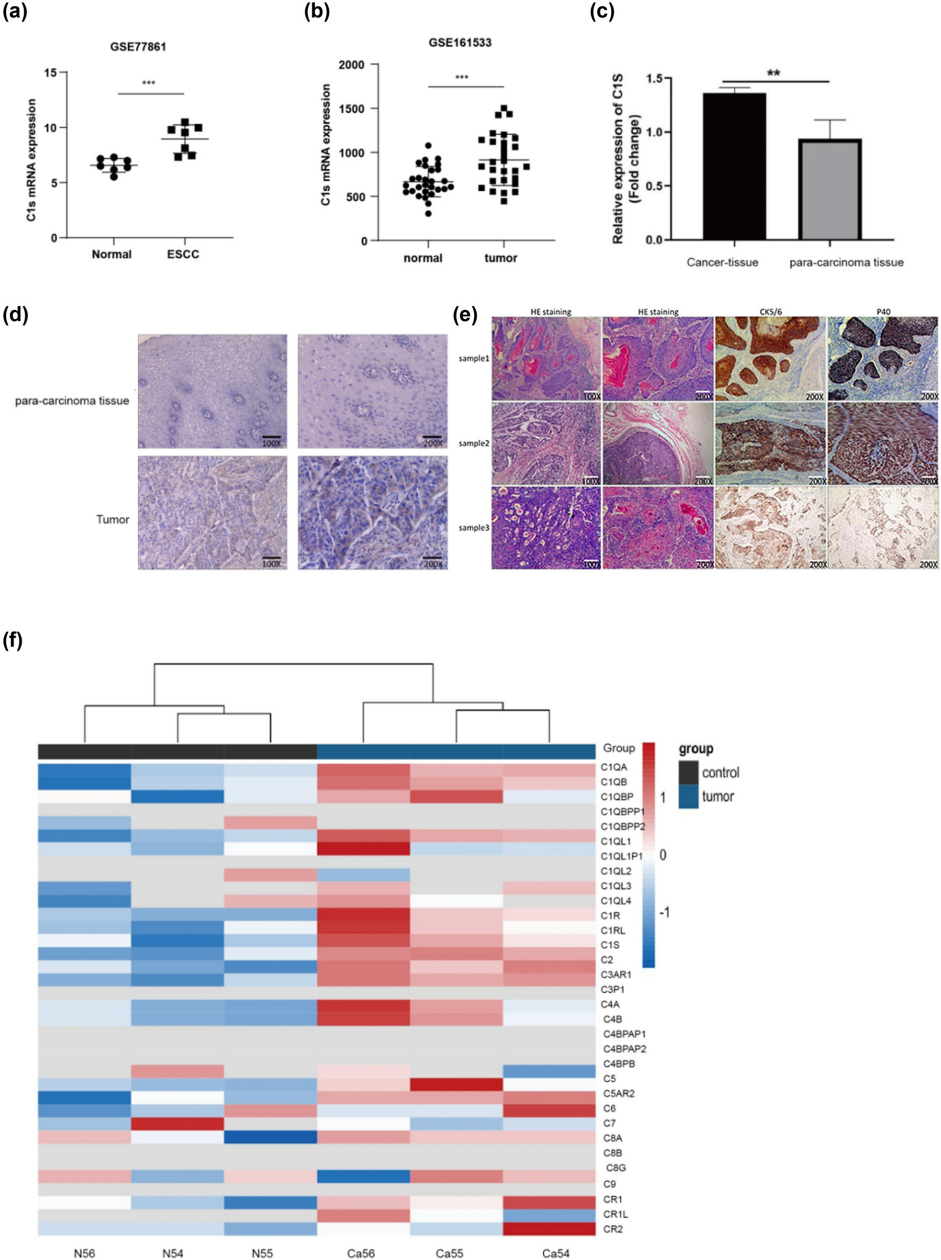
(a and b) Expression of C1s mRNA in ESCC and normal tissues in GSE77861 and GSE161533 datasets. (c) Expression of C1s in tumor tissues and adjacent tissues of ESCC patients. (d) IHC results of C1s in tumor tissue and paratumor tissues of ESCC patients (200×), (e) HE staining and IHC results of tumor tissue of three ESCC patients (200×). (f) Sequencing heat maps of tumor tissue and paratumor tissue of ESCC patients (**P < 0.05; ***P < 0.001).

As revealed by analysis based on Gene Expression Omnibus (GEO) datasets GSE77861 and GSE161533, C1s expression within ESCC tissues apparently increased relative to normal and adjacent non-cancerous tissues (*t* = 4.392, *P* < 0.01; *t* = 3.874, *P* < 0.01) (Figure 1a and b). This overexpression was confirmed by RT-qPCR (*P* < 0.01) (Figure 1c) and immunohistochemistry (*P* < 0.01) (Figure 1d and e). Besides, high-throughput sequencing of tumor and adjacent non-cancerous tissues in three ESCC patients also revealed significantly higher C1s expression in tumor tissues (*P* < 0.01) (Figure 1f).

### 3.2 Factors related to ESCC

Altogether 82 ESCC patients were examined, involving 70 males (85.4%) and 12 females (14.6%), with an average age of 58.28 ± 10.45 years. Significant factors related to ESCC were racial distinctions, residual lesions, and tumor site (*P* < 0.05), while age, T stage, and pathological grade did not show significant differences between tumor and non-cancerous tissues (Table 1).

**Table 1:** Baseline clinical data of ESCC patients

Project	*n*	Low expression of C1s	High expression of C1s	*P*
**Gender**				0.755
Male	70 (85.4%)	34 (41.5%)	36 (43.9%)	
Female	12 (14.6%)	7 (8.5%)	5 (6.1%)	
**Age**				1.000
≤60	53 (64.6%)	26 (31.7%)	27 (32.9%)	
>60	29 (35.4%)	15 (18.3%)	14 (17.1%)	
**T stage**				0.644
T1	8 (10.1%)	5 (6.3%)	3 (3.8%)	
T2	27 (34.2%)	11 (13.9%)	16 (20.3%)	
T3	41 (51.9%)	21 (26.6%)	20 (25.3%)	
T4	3 (3.8%)	1 (1.3%)	2 (2.5%)	
**T stage**				0.413
N0	46 (59.0%)	25 (32.1%)	21 (26.9%)	
N1	26 (33.3%)	10 (12.8%)	16 (20.5%)	
N2	5 (6.4%)	2 (2.6%)	3 (3.8%)	
N3	1 (1.3%)	1 (1.3%)	0 (0%)	
**Histological grading**				0.548
I	16 (11.3%)	5 (6.3%)	2 (2.5%)	
II	69 (48.7%)	23 (29.1%)	24 (30.4%)	
III	49 (34.5%)	9 (11.4%)	13 (16.5%)	
IV	8 (5.6%)	1 (1.3%)	2 (2.5%)	
**Race or ethnicity**				0.024
Asian	37 (46.3%)	15 (18.8%)	22 (27.5%)	
Black	6 (7.5%)	6 (7.5%)	0 (0%)	
White	37 (46.3%)	18 (22.5%)	19 (23.8%)	
**Residual lesion**				0.024
R0	65 (91.6%)	30 (42.3%)	35 (49.3%)	
R1	4 (5.6%)	4 (5.6%)	0 (0%)	
R2	2 (2.8%)	0 (0%)	2 (2.8%)	
**Tumor location**				0.030
Proximal esophagus	38 (47.0%)	22 (27.2%)	16 (19.8%)	
Mid esophagus	4 (45.7%)	19 (23.5%)	18 (22.2%)	
Distal esophagus	2 (7.4%)	0 (0%)	6 (7.4%)	

### 3.3 Knockdown of complement C1s significantly inhibits the proliferative ability of both normal esophageal epithelial and ESCC cells

**Figure 2:**
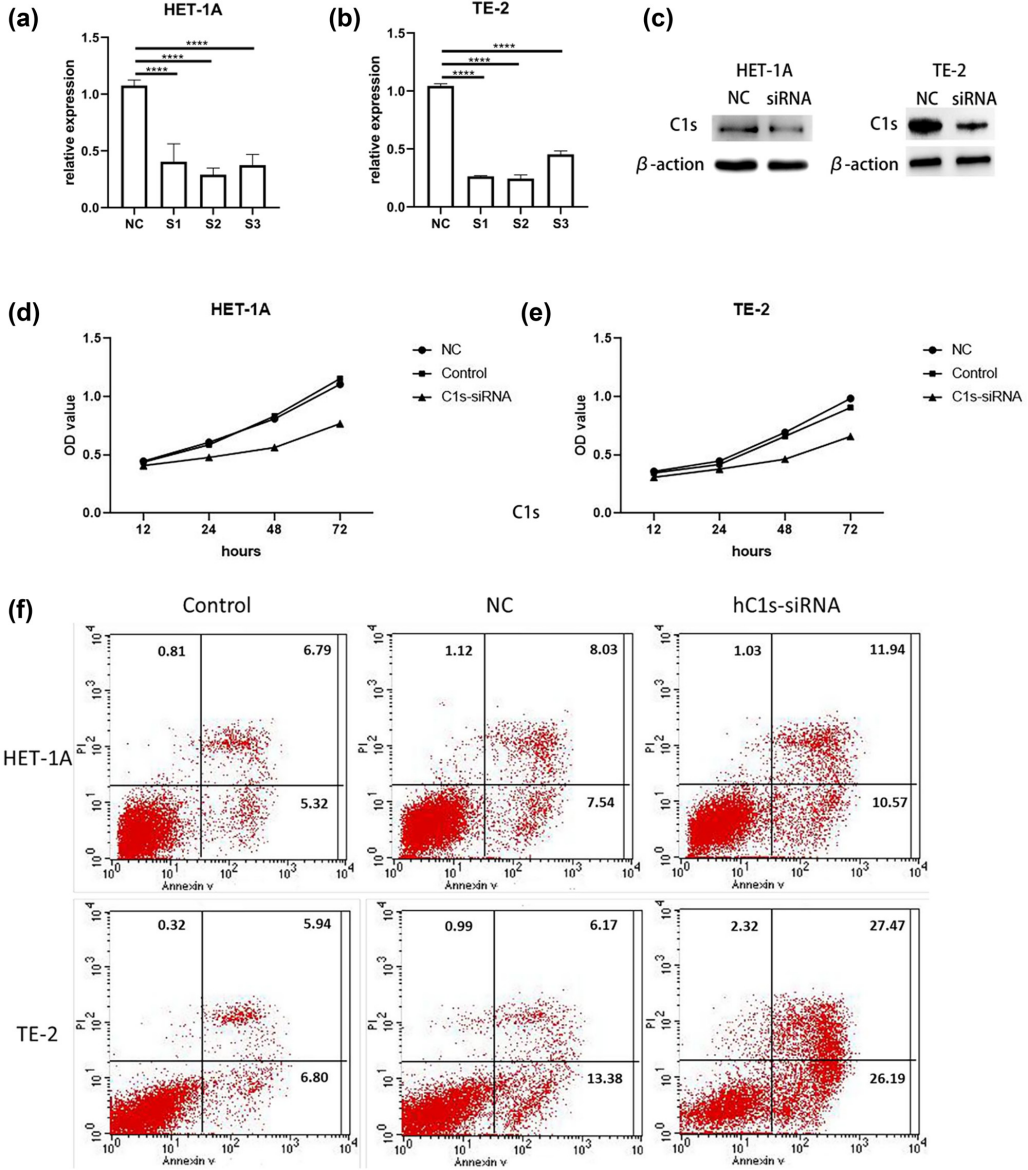
(a–c) Expression of HET-1A and TE-2 cells after C1s knockdown. (d and e) Complement C1s affected the proliferation of ET-1A and TE-2 cells. (f) Complement C1s affected apoptosis of ET-1A and TE-2 cells (****P < 0.0001).

Low C1s expression promoted apoptosis in both normal esophageal epithelial and ESCC cells, with a more pronounced effect being observed on ESCC cells. Transfection with siRNA significantly reduced the C1s mRNA expression (*P* < 0.0001), with hC1s si-2 showing the most effective knockdown effect on HET-1A and TE-2 cells (Figure 2a and b). Results of western blot assay confirmed that C1s protein expression significantly decreased (Figure 2c). CCK-8 assay indicated that C1s knockdown apparently inhibited cell proliferation (Figure 2d and e). Furthermore, flow cytometry analysis revealed that low C1s expression increased the apoptosis of both cell types, particularly in ESCC cells (Figure 2f).

### 3.4 Knockdown of C1s suppresses the expression of Wnt1/β-catenin pathway, which is associated with the proliferation of both HET-1A and TE-2 cells. Additionally, this knockdown influences the expression of cleaved-caspase3, Bax, and Bcl2, thereby promoting the apoptosis of HET-1A and TE-2 cells

**Figure 3:**
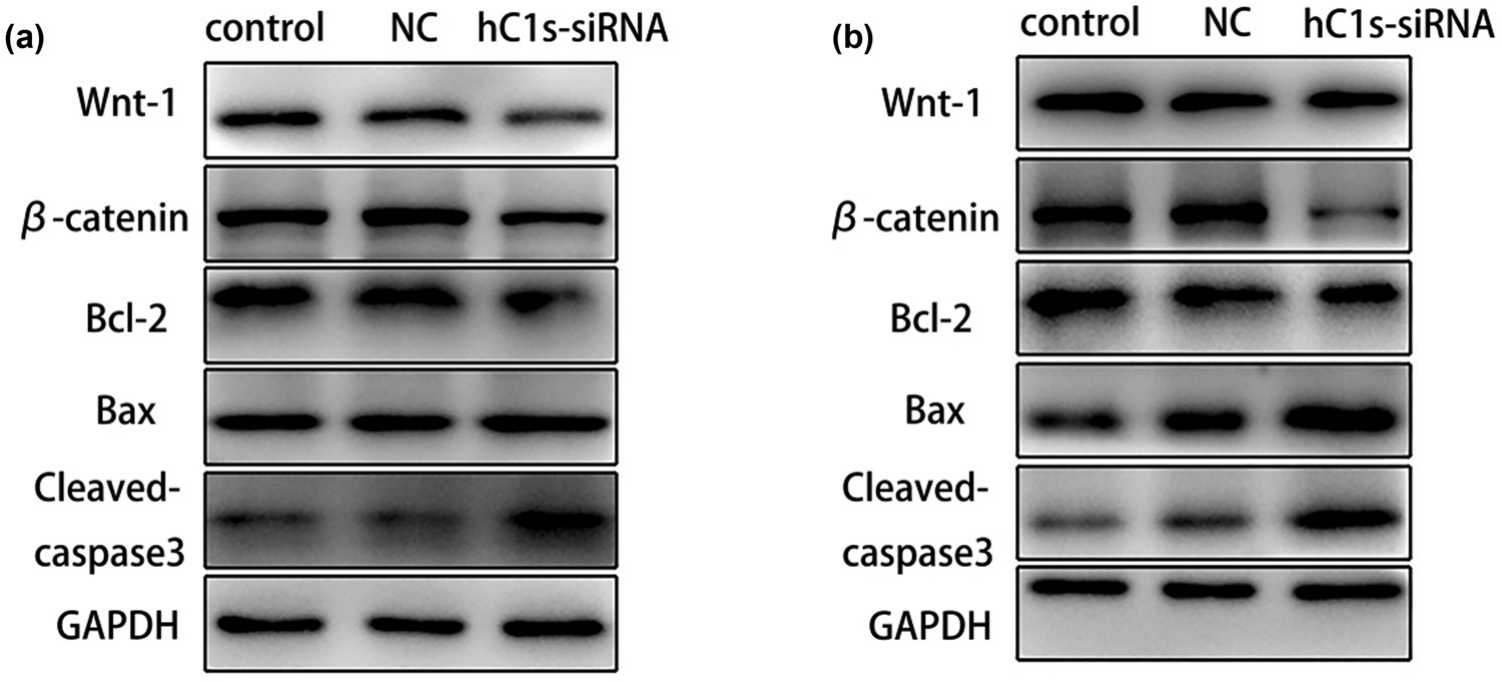
(a) Expression of proliferation and apoptosis-related molecules in HET-1A cells after C1s knockdown. (b) Expression of proliferation and apoptosis-related molecules in TE-2 cells after C1s knockdown.

According to previous studies, knockdown of C1s inhibits proliferation and promotes apoptosis in HET-1A and TE-2 cells; however, its specific mechanism remains unclear. As revealed by western blot, Wnt1 and β-catenin levels in HET-1A cells significantly decreased after the knockdown of C1s gene, indicating that C1s might inhibit cell proliferation via the Wnt1/β-catenin pathway. Additionally, in HET-1A cells with C1s gene knockdown, apoptosis-related protein Bcl2 had decreased expression, cleaved-caspase3 showed elevated expression, while Bax expression was not significantly changed. This suggested that C1s knockdown promoted cell apoptosis by down-regulating Bcl2 and up-regulating cleaved-caspase3 expression (Figure 3a). Similarly, in TE-2 cells, β-catenin expression significantly decreased after the knockdown of C1s gene, indicating that down-regulation of C1s inhibited cell proliferation by modulating the Wnt1/β-catenin pathway. Moreover, in TE-2 cells with C1s gene knockdown, Bcl2 expression decreased and Bax and cleaved-caspase3 increased, suggesting that C1s gene knockdown also promoted TE-2 cell apoptosis by down-regulating Bcl2 and up-regulating Bax and cleaved-caspase3 expression (Figure 3b).

## 4 Discussion

When screening for the high-risk populations of ESCC, blood testing holds significant potential [24]. As confirmed by our experimental results on the GEO databases GSE77861 and GSE161533, C1s expression increased in ESCC tumor tissues. Additionally, the expression of complement C1s in ESCC patients was examined through RT-PCR, immunohistochemistry, and high-throughput sequencing analysis, revealing the elevated C1s expression within ESCC tumor samples relative to adjacent non-cancerous tissues. Further, clinical data for ESCC patients were examined based on the TCGA database, which demonstrated a positive correlation between the C1s protein expression and patient factors such as race, residual lesions, and tumor location.

These findings suggest a correlation of C1s with ESCC, indicating its potential as a predictive marker for ESCC patients and a candidate indicator of tumor severity. Nonetheless, certain analyses are conducted on the basis of GEO and TCGA databases, and others have been validated in ESCC patients. Nevertheless, there is a lack of clinical information and follow-up records related to C1s expression in local patient tumor tissues, which may help further elucidate its clinical significance. As the influencing factors for ESCC include not only pathological elements but also non-pathological factors such as patients’ psychological well-being, compliance, and tumor sensitivity to radiotherapy and chemotherapy, further research is warranted to determine whether C1s affects the progression of ESCC via other pathways, such as regulating cellular growth functions.

Given the correlation of C1s expression with residual lesions and tumor location in ESCC, and considering the crucial importance of regulating tumor cell proliferation and apoptosis in cancer treatment, we proceeded to investigate the role of C1s in ESCC cell biological behavior. To this end, C1s expression was knocked down in human normal esophageal epithelial HET-1A cells and ESCC TE-2 cells. The C1s expression was intervened to observe its effects on proliferation and apoptosis in both normal esophageal epithelial and ESCC cells before and after intervention. This study aimed to shed more lights on the role of C1s in ESCC.

Proliferation and apoptosis not only play crucial roles in cell growth, but also make similar contributions to the occurrence and development of tumors [25]. As reported by research on U87 and T98G cell lines, knockdown of complement C1s significantly reduces cell viability and inhibits cell migration and invasion, indicating that complement C1s promotes glioblastoma cell proliferation [26]. Similarly, according to our results, C1s had a similar function in ESCC. After C1s down-regulation, alterations of cell proliferation were investigated by CCK-8 assays in HET-1A and TE-2 cells. As a result, the proliferative abilities of HET-1A and TE-2 cells significantly decreased after down-regulating C1s expression compared with the NC group, demonstrating that down-regulating C1s expression significantly inhibited the proliferation of the ESCC cell line TE-2.

As ESCC is usually diagnosed at the late stage, surgery may not be a viable treatment option, and the efficacy of adjuvant chemotherapy alone is not ideal and controversial. Therefore, in clinical practice, the majority of patients primarily undergo synchronous chemoradiotherapy for the treatment of ESCC [27,28]. Apoptosis, a crucial cell death mechanism, exerts a critical effect on radiation-mediated tumor killing [29]. In this study, after down-regulating C1s expression, flow cytometry analysis with Annexin V was conducted to detect the apoptosis of HET-1A and TE-2 cells. According to the results, the apoptosis of both HET-1A and TE-2 cells significantly increased after knockdown of C1s expression relative to the NC group. These findings suggested that down-regulating C1s expression markedly promoted the apoptosis of the ESCC cell line TE-2.

Further, the role of C1s in ESCC cell biological behavior was explored by examining factors affecting cell proliferation and apoptosis of human normal esophageal epithelial HET-1A cells and ESCC TE-2 cells with C1s knockdown. This allowed us to delve into the associated molecular mechanisms within ESCC.

Interaction of the Wnt/β-catenin signaling pathway exerts a crucial effect on maintaining intracellular homeostasis, besides, it acts as a key factor for the development and progression of tumors [30,31]. Studies on transgenic mouse models have demonstrated that inhibiting the expression of Wnt1 alleviates the growth and progression of breast cancer [32,33]. In terms of treatment, blocking Wnt1 has been observed to induce apoptosis of colorectal cancer cells [34]. Actually, some clinical trials have assessed drugs that inhibit Wnt/β-catenin pathway as the potential way to produce the anti-tumor effects. Considering the significance of Wnt/β-catenin pathway for the link between precancerous lesions and cancer, this study further analyzed whether C1s affected the proliferation of ESCC via the Wnt1/β-catenin pathway. In this study, C1s affected ESCC cell proliferation by modulating Wnt1/β-catenin cellular pathway.

Cell apoptosis has an important part in the progression of tumors, and apoptotic factors such as Bax, Bcl-2, and caspase-3 have been extensively studied [35]. Based on our observations, C1s influenced the ESCC cell apoptosis through regulating levels of apoptotic factors Bcl2, Bax, and cleaved-caspase3, thereby affecting ESCC genesis and progression. Given the propensity of ESCC for metastasis, our future research will focus on the role of C1s in ESCC cell invasion and migration.

Taken together, by employing bioinformatics methods in conjunction with *in vitro* cell experiments, the differential expression and influencing factors for C1s in ESCC were investigated. This study provides an initial exploration of the C1s biological effect on ESCC progression, which lays a theoretical foundation for further in-depth research. Subsequent studies will delve into the specific molecular mechanisms and pathways of C1s in ESCC.
